# Visualization of Laterally Spreading Colorectal Tumors by Using Image-Enhanced Endoscopy

**DOI:** 10.1155/2012/638391

**Published:** 2012-01-18

**Authors:** Naoto Tamai, Yutaka Saito, Taku Sakamoto, Takeshi Nakajima, Takahisa Matsuda, Namasivayam Vikneswaran, Hisao Tajiri

**Affiliations:** ^1^Endoscopy Division, National Cancer Center Hospital, 5-1-1 Tsukiji, Chuo-ku, Tokyo 104-0045, Japan; ^2^MBBS, Department of Gastroenterology and Hepatology, Singapore General Hospital, Singapore 769608; ^3^Department of Gastroenterology and Hepatology, The Jikei University School of Medicine, 3-25-8 Nishi Shinbashi, Minato-ku, Tokyo 105-8461, Japan

## Abstract

Laterally spreading tumors may sometimes evade detection by colonoscopy. This study aimed to evaluate the use of image-enhanced endoscopy for visualizing laterally spreading tumors of the nongranular type. We reviewed consecutive patients with 47 non-granular-type laterally spreading tumors that had been examined using white-light imaging, autofluorescence imaging, narrow-band imaging, and chromoendoscopy with indigo carmine. The quality of visualization was evaluated using a 5-point scale by less- and more-experienced endoscopists. Autofluorescence imaging provided significantly better visualization than white-light imaging for both less-experienced and experienced endoscopists. On the other hand, no significant differences were observed between the quality of visualization provided by white-light imaging and narrow-band imaging for less-experienced endoscopists. Autofluorescence imaging provides high-quality visualization of non-granular-type laterally spreading tumors on still images. Multicenter trials should be conducted to confirm the usefulness of autofluorescence imaging in detecting laterally spreading colorectal tumors.

## 1. Introduction

Colorectal carcinoma is one of the most common cancers worldwide, and its prevalence is steadily increasing in Japan [1]. Colonoscopy is considered the gold standard for the detection of neoplastic lesions at risk of progression to colorectal carcinoma. However, according to the results of back-to-back colonoscopies by Rex et al., the miss rate for adenomas ≥1 cm was 6% [2]. Laterally spreading tumors (LSTs) constitute a subset of nonpolypoidal colonic lesions, which are characterized by lateral and circumferential extension along the colonic wall rather than vertical growth [3]. LSTs are further classified based on their macroscopic appearance. The granular type LST (LST-G) is defined by the presence of aggregates of even or uneven nodules on the surface, whereas the non-granular-type LST (LST-NG) has a smooth surface lacking the granulonodular formations [4, 5]. Owing to the flat shape of LSTs, the miss rate for these tumors might be higher than the 6% reported by Rex et al. In addition, LSTs, particularly the NG type, have a higher potential for malignancy; nearly 30% of LST-NGs are associated with lymph follicular or multifocal submucosal invasion [6]. A reduction in the miss rate for LST-NG could therefore contribute to colorectal cancer prevention. Emerging data suggest that the use of image-enhanced endoscopy (IEE) such as autofluorescence imaging (AFI) and narrow-band imaging (NBI) may lead to improvements in polyp detection rates, although this notion remains controversial [7–15]. In our experience, we have encountered many LST-NG lesions that were better visualized by IEE than by white-light imaging (WL). The aim of this study was to evaluate the quality of visualization of LST-NG provided by IEE.

## 2. Methods

From September 2009 to April 2011, consecutive patients with LST-NG lesions resected by endoscopic submucosal dissection (ESD) in our institution were included in this study. The inclusion criteria for performing ESD on LST-NGs were as follows: (1) evidence of a noninvasive pattern [15–17] and (2) lesions larger than 20 mm that were difficult to resect enbloc by using conventional EMR [18]. First, endoscopic examinations were performed using the white-light mode of the AFI videoendoscope system to identify LST-NG lesions, once lesions were detected, the colonoscopist conducted AFI and NBI examinations by switching first to the AFI mode followed by the NBI mode, and finally lesions were examined by chromoendoscopy (CE) using the white-light mode. AFI colonoscopes (EVIS CF-FH260AZI; Olympus Medical Systems, Tokyo, Japan), light sources (EVIS CLV-260SL; Olympus Medical Systems), and video processors (EVISLUCERA CV-260SL; Olympus Medical Systems) were used in this study. The AFI videoendoscope system is a novel illumination method that produces real-time pseudocolor images. Neoplastic lesions involve a thickening of the mucosal layer and increased hemoglobin so such lesions emit weaker autofluorescence compared to nonneoplastic lesions; therefore nonneoplastic lesion appears green, while neoplastic lesion has a magenta image [7]. The AFI system allowed for immediate switching from WL to AFI and NBI with a button on the control head of the endoscope. CE was performed using 0.4% indigo carmine. Images of the lesions from WL, NBI, AFI, and CE without magnification were captured and electronically archived in the electronic medical records of our hospital. The images were selected by an experienced endoscopist blinded to this study. The WL, NBI, AFI, and CE images for each lesion were downloaded. The images of all the lesions were randomly arranged, and a Microsoft PowerPoint presentation was created. These images did not contain any information to identify the patient or the lesion. The PowerPoint presentations were sent to the respective raters for their independent evaluation. The images were assessed by 2 groups of endoscopists (A and B). Group A comprised 2 physicians with no previous experience in IEE, and group B comprised 2 endoscopists, each of whom had analyzed over 100 cases by using IEE. Each endoscopic image was assessed and given a global rating for visualization based on the ability to detect the lesion and the clarity of the tumor margins. The images were rated by the endoscopists on a 5-point scale as follows: 5, very well visualized; 4, well visualized; 3, moderately well visualized; 2, poorly visualized; 1, very poorly visualized. The ratings of the images were analyzed separately for groups A and B. For each group of raters, the quality of visualization of lesions that received a score of 4 or more from both the raters was classified as “good”. The quality of visualization of lesions with a score below 4 was classified as “poor.” 

## 3. Statistical Analysis

Statistical analysis was performed using SPSS for Windows (SPSS, Release 6.0; SPSS Inc., Chicago, Ill, USA, 1993). Statistical significance was defined as a *P*-value less than 0.05.

## 4. Results

**Figure 1:**
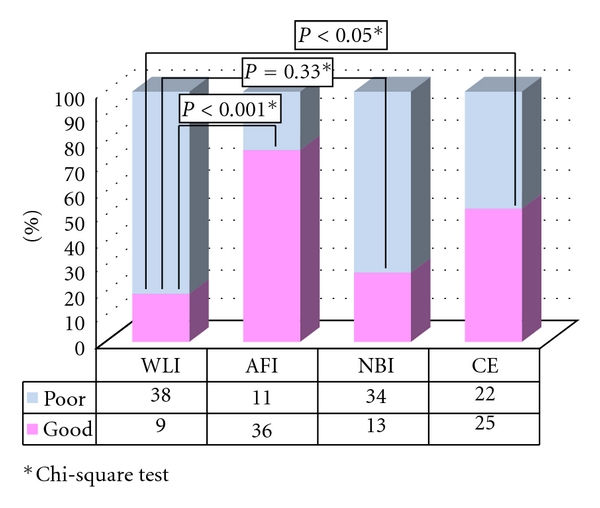
Visualization of LST-NG in group A.

In all, 49 LST-NG lesions in 47 patients were included in this study. Two patients with lesions were excluded from this study, because the lesions were not observed in the same field in each of the 4 modalities. Finally, a total of 47 LST-NG lesions in 45 patients were evaluated (Table 1). Of the 47 lesions analyzed in group A, the quality of visualization was categorized as “good” for 6 lesions using AFI, 13 using NBI, and 25 using CE. AFI (36/47) provided significantly better visualization than WL (9/47) (*P* < 0.001). Similarly, there was a significant difference between the quality of visualization using CE (25/47) and WLI (9/47) (*P* < 0.05). There was no significant difference, however, between WLI (9/47) and NBI (25/47) (Figure 1). Regarding AFI visualization, there was no significant difference in the macroscopic subtype, tumor location, or underlying histology between well-visualized and poorly visualized lesions, but well-visualized lesions were larger than the poorly visualized lesions (Table 2).

**Figure 2:**
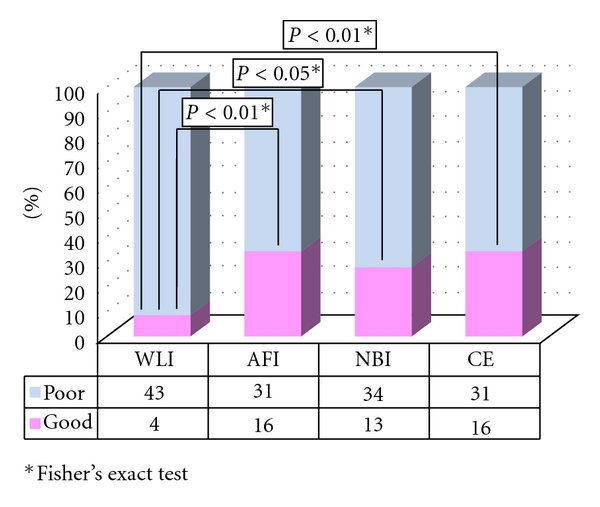
Visualization of LST-NG in group B.

In group B, the quality of visualization was assessed as “good” for 4 lesions by using WLI, 16 lesions by using AFI, 13 lesions by using NBI, and 16 lesions by using CE. There was a significant difference in the frequency of well-visualized lesions between AFI (16/47) and WLI (4/47) (*P* < 0.001). Similarly, a significant difference in visualization quality was observed between CE (16/47) and WLI (16/47) (*P* < 0.01) and between NBI (13/47) and WLI (4/47) (*P* < 0.05) in group B (Figure 2). Regarding AFI, there was no significant difference in the macroscopic subtype, tumor location, or underlying histology between well-visualized and poorly visualized lesions. Well-visualized lesions were larger than the poorly visualized ones (Table 3).

## 5. Discussion

**Figure 3:**
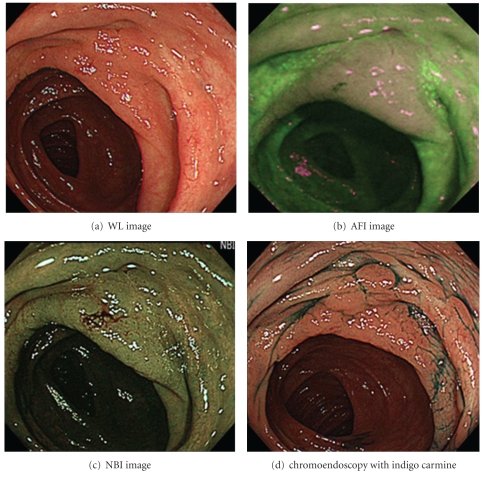
LST-NG lesions categorized as “wellvisualized” using AFI. Location: Transverse colon. Size of the lesion: 45 mm. Macroscopic type: IIa (LST-NG). Pathological findings: well-differentiated adenocarcinoma, low-grade atypia, Pm.

**Figure 4:**
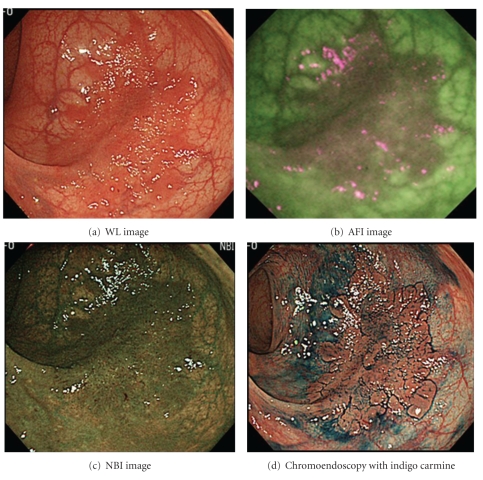
LST-NG lesions categorized as “wellvisualized” by using AFI. Location: lower rectum. Size of the lesion: 45 mm. Macroscopic type: IIa (LST-NG). Pathological findings: well and moderately differentiated adenocarcinoma, pSM (350 um).

Based on the results of our study, AFI provides good-quality visualization of LST-NG lesions, not only for experienced endoscopists but for less-experienced endoscopists as well. The utility of AFI for the detection of colorectal tumors still remains controversial, with studies reporting mixed results [7–9, 15, 19]. In this study, 2 LST-NG lesions were determined to be well visualized by 4 endoscopists (Figures 3 and 4.). As Figures 1 and 2 show, we observed LST-NG lesions that were better visualized using AFI than the other methods. The relationship between visualization and detection is uncertain. However, better visualization may enable improved detection of LST lesions, especially those of the NG type, which have been shown to be difficult to detect with CE [4].  It is particularly important to improve the detection rate of LST-NGs, because they are more likely to harbor malignancy; nearly 30% of LSTs of the NG type involve lymph follicular or multifocal submucosal invasion [6]. Though LST-NG lesions are less prevalent than polypoidal lesions, their greater malignant potential necessitates reliable detection methods. This study suggests that AFI is superior to WLI for the detection of LST-NG lesions at least on still images. In the present study, there was no significant difference in the quality of visualization of LST-NGs between WLI and NBI for the less-experienced endoscopists.

**Figure 5:**
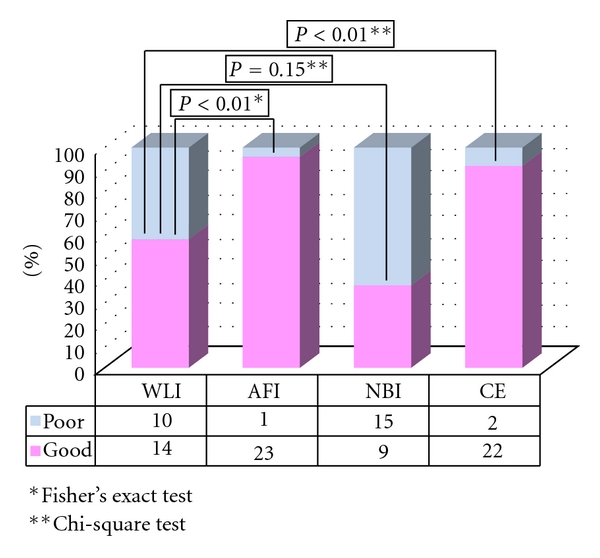
Visualization of LST-G in group A.

**Figure 6:**
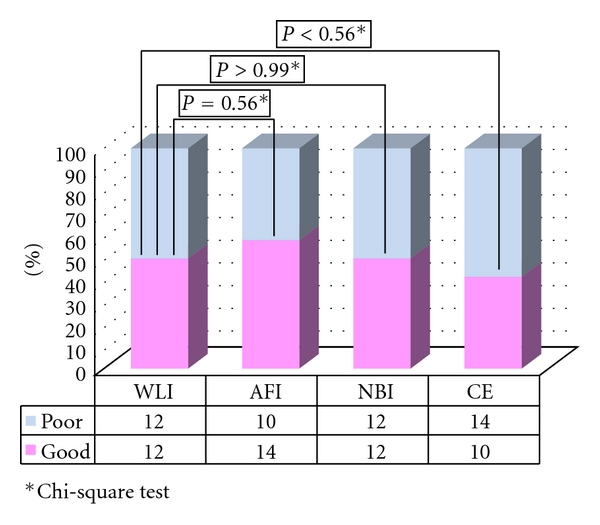
Visualization of LST-G in group B.

We also evaluated LST-G lesions in the same fashion as for the LST-NGs. As shown in Figures 5 and 6, AFI also provided good-quality visualization of LST-G lesions for the less-experienced endoscopists, despite the lack of a significant difference in visualization quality between WLI and AFI for the experienced endoscopists. This result indicates that an advantage of AFI might be that it simplifies observations for less-experienced endoscopists. We also compared the backgrounds of the LST-NG lesions between those with good versus poor visualization quality by using AFI. There were no significant differences between lesions that had good versus poor visualization quality with respect to macroscopic type, location, or pathological findings. However, the well-visualized lesions were larger than the poorly visualized lesions in groups A and B. To obtain a whole image of a large lesion, it is necessary to maintain sufficient distance between the tip of the scope and the lesion, which may affect the visibility of the lesion.

This study had several limitations. Only still images were evaluated, and it is uncertain if these findings can be applied to real-time video endoscopy. A relatively small sample precludes any multivariate analysis. Larger studies are needed to define the factors influencing the quality of visualization. 

## 6. Conclusion

AFI provides good-quality visualization of LST-NG lesions on still images. However, to confirm the detectability of LST-NG lesions by using AFI, multicenter trials should be performed.

## Figures and Tables

**Table 1:** Characteristics of lesions.

Number of lesions	47
Number of patients	45
Sex	
Male	31
Female	24
Age (years)	
Median	69
Range	50–80
Tumor size (mm)	
Median	30
Range	20–60
Tumor location	
Cecum	1
Colon	39
Rectum	7
Histopathology	
Adenoma	5
m-ca	24
sm superficial (sm1^*∗*^)	11
sm deep (sm2-3)	7

*sm1 : sm < 1000 *μm*.

**Table 2:** Backgrounds of the LST-NG lesion evaluated by AFI in group A.

	Quality of visualization		
	Good	Poor	*P*
Macroscopic type			
Flat elevated	32	9	0.30*
Flat or flat depressed	4	2	
Lesion size (mm)			
Median	25	35	<0.05**
Range	20–50	20–60	
Location			
Rectum	6	1	0.34*
Cecum or colon	30	10	
Pathological finding			
Adenoma	4	2	0.30*
Adenocarcinoma	32	9	

*Fisher's exact test.

**Mann-Whitney test.

**Table 3:** Characterization of LST-NG lesions by AFI in group B.

	Quality of visualization		
	Good	Poor	*P*
Macroscopic type			
Flat elevated	16	25	0.07*
Flat or flat depressed	0	6	
Lesion size (mm)			
Median	25	30	<0.05**
Range	20–45	20–60	
Location			
Rectum	2	5	0.32*
Cecum or colon	14	26	
Pathological finding			
Adenoma	4	2	0.08*
Adenocarcinoma	12	29	

*Fisher's exact test.

**Mann-Whitney test.
